# Routine Check-Ups and Other Factors Affecting Discussions With a Health Care Provider About Subjective Memory Complaints, Behavioral Risk Factor Surveillance System, 21 States, 2011

**DOI:** 10.5888/pcd13.150471

**Published:** 2016-01-28

**Authors:** Mary Adams

## Abstract

**Introduction:**

Most adults reporting subjective memory complaints (SMCs) do not discuss them with a health care provider and miss an opportunity to learn about treatment options or receive a diagnosis. The objective of this study was to describe correlates of discussing memory problems with a health care professional among adults reporting SMCs.

**Methods:**

Data were from 10,276 respondents aged 45 years or older in 21 states reporting SMCs on the 2011 Behavioral Risk Factor Surveillance System (BRFSS). Odds ratios (ORs) adjusted for demographic and health-related measures were computed for discussing SMCs with a health care professional.

**Results:**

Among all respondents aged 45 or older reporting SMCs, 22.9% reported discussing them with a health care professional; among those reporting a recent routine check-up, this rate was 25.2%. The largest adjusted OR for discussing SMCs with a health care professional was for respondents reporting that SMCs always (vs never) caused them to give up household chores (OR, 3.02) or always (vs never) interfered with work (OR, 2.98). Increasing age reduced the likelihood of discussing SMCs. Among respondents who discussed SMCs, 41.8% received treatment.

**Conclusion:**

Routine check-ups may be a missed opportunity for discussions of SMCs that might lead to diagnosis or treatment. The Affordable Care Act requires a cognitive assessment for Medicare recipients during their annual wellness visit, but these results suggest that adults younger than 65 might also benefit from such an assessment.

## MEDSCAPE CME

Medscape, LLC is pleased to provide online continuing medical education (CME) for this journal article, allowing clinicians the opportunity to earn CME credit.

This activity has been planned and implemented in accordance with the Essential Areas and policies of the Accreditation Council for Continuing Medical Education through the joint sponsorship of Medscape, LLC and *Preventing Chronic Disease*. Medscape, LLC is accredited by the ACCME to provide continuing medical education for physicians.

Medscape, LLC designates this Journal-based CME activity for a maximum of 1 ***AMA PRA Category 1 Credit(s)™***. Physicians should claim only the credit commensurate with the extent of their participation in the activity.

All other clinicians completing this activity will be issued a certificate of participation. To participate in this journal CME activity: (1) review the learning objectives and author disclosures; (2) study the education content; (3) take the post-test with a 75% minimum passing score and complete the evaluation at www.medscape.org/journal/pcd; (4) view/print certificate.

**Release date: January 28, 2016; Expiration date: January 28, 2017**

### Learning Objectives

Upon completion of this activity, participants will be able to:

1. Assess the percentage of older adults with subjective memory complaints who discussed memory problems with their clinician, based on a surveillance study; correlates of having such discussions; and clinical implications of these findings
2. Identify correlates of older adults with subjective memory complaints having discussions with their clinician about their memory problems
3. Determine the clinical implications of findings from this surveillance study regarding older adults with subjective memory complaints discussing their memory problems with their clinician

**EDITOR**

Ellen Taratus, Editor, *Preventing Chronic Disease*. 

Disclosure: Ellen Taratus has disclosed no relevant financial relationships.

**CME AUTHOR**

Laurie Barclay, MD, Freelance writer and reviewer, Medscape, LLC

Disclosure: Laurie Barclay, MD, has disclosed no relevant financial relationships.

**AUTHOR**

Mary Adams, MS, MPH, On Target Health Data LLC, West Suffield, Connecticut

Disclosure: Mary Adams, MS, MPH, has disclosed no relevant financial relationships.

## Introduction

Although the US Preventive Services Task Force (1) does not recommend routine screening for dementia, which is often first manifested in memory problems, early diagnosis is important for numerous reasons. These include ruling out treatable causes of memory problems not related to dementia, providing a better chance for treatment to be effective, offering enrollment in clinical trials, and discussing community services (2–4). Another important consideration is identifying memory problems at a stage when the individual is still able to participate in decision making about his or her own future. The Affordable Care Act requires that Medicare recipients receive a cognitive assessment during their covered annual wellness visit (5), and recommendations for tools to use during this assessment are available (6).

The Healthy Aging Program at the Centers for Disease Control and Prevention (CDC) developed an optional module for use on the Behavioral Risk Factor Surveillance System (BRFSS) that addresses cognitive health and decline (7–10). The first question addresses confusion or memory loss that is happening more often or is getting worse during the past 12 months, defined hereinafter as subjective memory complaints (SMCs). Follow-up BRFSS questions address the need for help, functional difficulties, whether the respondent discussed the increased confusion or memory loss with a health care professional, and if so, whether or not treatment was received. Discussions with a health care professional about memory problems could serve some of the same purposes as assessment by providing an opportunity to learn about services and treatment. This study was conducted to determine how many adults reporting SMCs discussed their memory problems with a health care professional, the factors associated with that action, and the proportion who received treatment. Of special interest were respondents reporting a routine check-up within the previous year.

## Methods

Data were from publicly available (11) BRFSS data collected in 2011 by 21 states (Arkansas, California, Florida, Hawaii, Iowa, Illinois, Louisiana, Maryland, Michigan, North Carolina, Nebraska, New Hampshire, New York, Oklahoma, South Carolina, Tennessee, Texas, Utah, Washington, Wisconsin, West Virginia) that included the cognitive impairment module (7). The BRFSS collects data from noninstitutionalized adults aged 18 or older through monthly random-digit–dial telephone surveys (12) and uses a complex sample design to randomly select respondents with either landline or cellular telephones. Data are weighted to account for the probability of selection of respondents and are further adjusted to be representative of the total adult population of each state by age, race/ethnicity, sex, marital status, education, home ownership, and type of telephone service. Data for this study were limited to the 121,304 landline surveys and excluded cellular telephone surveys, which were available for only 7 of the 21 states. Because 9 states used the cognitive impairment module on split surveys, data from different survey versions were combined and the weighting variables were renamed to be the same, as described by CDC (13). The median survey response rate for landline surveys in the 21 states in 2011 was 53.4%, ranging from 37.4% in California to 66.0% in Nebraska (14).

Respondents answering yes to the question “During the past 12 months, have you experienced confusion or memory loss that is happening more often or is getting worse?” were defined as reporting SMCs. Based on preliminary results of SMCs by age group for ages 18 to 24 (5.3%), 25 to 34 (6.9%), 35 to 44 (7.5%), 45 to 54 (12.2%), 55 to 64 (12.2%), and 65 or older (13.0%), data were restricted to the 95,147 respondents aged 45 or older. This group included 86.7% of all respondents reporting SMCs (10,583 of 12,205); the same age cutoff was used for these data in another study (10). A follow-up question for those reporting SMCs asked about the areas in which they need the most assistance as a result of their confusion or memory loss. Possible answers were 1) safety, 2) transportation, 3) household activities, 4) personal care 5) need assistance but not in those areas, or 6) no assistance needed in any area. Responses were dichotomized into need help or not. Respondents with functional difficulties were determined from responses to 2 questions and included those who responded that, during the previous 12 months, they always, usually, or sometimes (as opposed to rarely or never) gave up household activities or chores they used to do because of SMCs; or SMCs always, usually, or sometimes (as opposed to rarely or never) interfered with their ability to work, volunteer, or engage in social activities; or both. These measures were also each used separately in logistic regression as 5-level measures, with never as the referent. Additional questions asked whether their confusion or memory loss was discussed with a health care professional (yes/no), whether they received treatment such as therapy or medications for confusion or memory loss (yes/no), and whether a health care professional ever said they had Alzheimer’s disease or some other dementia (answers of yes to Alzheimer’s disease or other dementia were combined vs no diagnosis). Respondents who had not discussed their SMCs with a health care provider were not asked about treatment or diagnosis, so those records were coded as no.

Other measures included age (45–54, 55–64, 65–74, 75–84, and ≥85 y), sex, education (<high school, high school graduate, some college, and college graduate), annual income (<$15,000, $15,000–$24,999, $25,000–$49,999, $50,000–$74,999, and ≥$75,000), race/ethnicity (non-Hispanic white, non-Hispanic black or African American, Hispanic of any race, and other), disability (limited in any way in any activities or use special equipment [yes/no]), ever diagnosed with depression (yes/no), health status (excellent, very good, or good vs fair or poor), reporting any of 6 chronic diseases (arthritis, asthma, cardiovascular disease, cancer other than skin, chronic obstructive pulmonary disease, or diabetes [yes/no]), weight status (body mass index <18.5, 18.5–24.9, 25.0–29.9, 30.0–34.9, and ≥35.0), reporting a cost barrier to health care (Was there a time in the past 12 months when you needed to see a doctor but could not because of cost? [yes/no]), having one or more personal doctors (yes/no), health insurance (yes/no), reporting a routine check-up within the previous year (yes/no) (hereinafter referred to as a recent check-up), current smoking (yes/no), and state of residence.

Stata version 14.0 (StataCorp LP) was used for all analysis to account for the complex sample design of the BRFSS; analysis used the landline weight or the corresponding weight for states that had multiple survey versions. Pearson χ^2^ tests of association (α = .05) from cross-tabulations of talking with a health care professional about their SMCs and demographic and health-related measures were used to select variables to include in the multivariate logistic regression model. Adjusted odds ratios (ORs) and 95% confidence intervals (CIs) are reported for the final logistic regression model after removal of variables that had *P* values >.05. For all measures, refusal to answer or responses of “don’t know” were excluded. Excluding respondents with missing values for having a discussion with a health care professional reduced the sample size from 10,583 to 10,276.

## Results

One in 8 respondents age 45 or older (12.5%; 95% CI, 12.0%–13.0%) reported SMCs, with minor (but significant) variation (11.9%–15.6%) across the 5 age groups. Compared with respondents aged 45 or older not reporting SMCs, those reporting SMCs were more likely to live in California (22.4% vs 17.1%); be Hispanic (14.4% vs 11.2%), aged 75 or older (17.6% vs 15.0%), and currently smoke (24.5% vs 15.2%); they were less likely to be white (68.1% vs 72.5%). They also reported less education and lower income, whereas sex distribution was similar for both groups.

Among the 10,276 respondents aged 45 or older reporting SMCs, 22.9% (95% CI, 21.1%–24.9%) reported discussing their memory problems with a health care professional. Significant differences in the percentages of adults who talked to a health care professional about their SMCs are shown in Table 1. Of adults aged 45 or older reporting SMCs, the three-fourths who had a recent check-up were more likely than those who did not have a recent check-up to discuss their SMCs with a health care professional, but still nearly 75% had not discussed their memory issues with a health care professional. Adults reporting functional difficulties due to their SMCs were significantly more likely than those without functional difficulties to have talked with a health care professional about their memory problems. Among those with SMCs and functional difficulties, those who had a recent check-up were more likely than those not having one to have talked with a health care professional (37.4% [95% CI, 33.2%–41.7%] vs 25.2% [95% CI, 19.8%–31.5%]). Sex, race/ethnicity, income, insurance status, and reporting a cost barrier to health care were not significantly associated with talking with a health care professional so were excluded from Table 1 and the logistic regression model. 

**Table 1:** Self-Reported Discussions With a Health Care Professional About Memory Problems, Adults Aged ≥45 Years Reporting Subjective Memory Complaints, by Selected Characteristics^a^, BRFSS in 21 states, 2011

Characteristic	No. of Respondents	Respondents Who Reported Discussion, % (95% CI)
**All adults aged ≥45 with SMCs**	10,276	22.9 (21.1–24.9)
**Age, y**		
45–54	2,175	27.1 (23.3–31.2)
55–64	2,948	24.8 (21.6–28.3)
65–74	2,439	18.0 (15.2–21.2)
75–84	2,008	16.8 (13.7–20.4)
≥85	706	14.4 (10.3–19.7)
*P* value	<.001	
**Education**		
<High school	1,523	19.2 (15.6–23.5)
High school graduate	3,414	21.9 (18.6–25.5)
Some college	2,790	26.2 (22.7–30.1)
College graduate	2,529	24.4 (21.2–27.8)
*P* value	.04	
**Disability^b^**		
Any disability	6,713	29.2 (26.8–31.8)
No disability	3,513	11.4 (9.3–13.8)
*P* value	<.001	
**Body mass index, kg/m^2^**		
<18.5	202	24.6 (13.5–40.4)
18.5–24.5	2,932	20.2 (17.3–23.5)
25.0–29.9	3,401	20.4 (17.5–23.7)
30.0–34.9	1,950	23.5 (19.6–27.8)
≥35.0	1,469	31.5 (26.0–37.5)
*P* value	.002	
**Any of 6 chronic diseases^c^**		
Yes	8,365	24.5 (22.4–26.7)
No	1,809	17.0 (13.3–21.5)
*P* value	.004	
**Current smoking**		
Yes	2,054	27.1 (23.1–31.5)
No	8,174	21.7 (19.7–23.8)
*P* value	.02	
**Had a routine check-up within the previous year**		
Yes	7,909	25.2 (23.0–27.5)
No	2,191	16.2 (13.3–19.6)
*P* value	<.001	
**Has ≥1 personal doctors**		
Yes	9,493	24.3 (22.4–26.3)
No	753	11.3 (7.7–16.4)
*P* value	<.001	
**Health status**		
Fair or poor	4,928	28.2 (25.4–31.2)
Excellent, very good, or good	5,309	17.6 (15.4–20.2)
*P* value	<.001	
**Ever diagnosed with depression**		
Yes	4,254	33.2 (29.9–36.7)
No	5,944	14.6 (12.8–16.6)
*P* value	<.001	
**Need help because of SMCs**		
Yes	5,324	29.0 (26.3–31.8)
No	4,640	15.4 (13.1–17.9)
*P* value	<.001	
**Gave up household activities or chores**		
Never	5,632	14.5 (12.7–16.5)
Rarely	1,608	24.3 (20.1–29.0)
Sometimes	2,104	32.1 (27.2–37.3)
Usually	428	41.9 (31.1–53.4)
Always	359	54.1 (43.8–64.1)
*P* value	<.001	
**SMCs interfere with working, volunteering, or engaging in social activities**		
Never	5,183	12.5 (10.7–14.6)
Rarely	1,859	21.4 (17.8–25.6)
Sometimes	1,886	33.8 (29.1–38.9)
Usually	499	38.9 (29.7–48.9)
Always	728	51.3 (43.1–59.4)
*P* value	<.001	
**Functional difficulties^d^**		
Yes	3,995	34.4 (30.9–38.0)
No	6,205	14.5 (12.7–16.4)
*P* value	<.001	
**State of residence**		
Arkansas	531	22.2 (17.7–27.5)
California	498	14.8 (11.6–18.7)
Florida	970	25.2 (20.1–31.1)
Hawaii	483	24.4 (18.0–32.3)
Illinois	347	24.7 (18.2–32.6)
Iowa	356	22.4 (17.3–28.5)
Louisiana	499	32.4 (24.9–41.0)
Maryland	281	18.8 (13.7–25.2)
Michigan	310	24.4 (15.9–35.5)
Nebraska	858	18.2 (14.8–22.3)
New Hampshire	277	28.7 (21.7–36.9)
New York	210	25.3 (18.4–33.8)
North Carolina	661	25.2 (20.7–30.2)
Oklahoma	335	25.3 (20.1–31.2)
South Carolina	941	20.9 (16.4–26.2)
Tennessee	248	33.4 (26.1–41.6)
Texas	581	31.9 (25.4–39.1)
Utah	242	20.9 (14.9–28.6)
Washington	1,036	19.6 (16.6–23.1)
West Virginia	278	32.0 (25.8–38.8)
Wisconsin	334	18.4 (12.9–25.7)
*P* value	<.001	

Abbreviations: BRFSS, Behavioral Risk Factor Surveillance System; CI, confidence interval; SMC, subjective memory complaint.

a Characteristics are those that had *P* values <.05 in Pearson χ^2^ tests of association; these variables were included in the starting logistic regression model. Sex, race/ethnicity, income, insurance status, and reporting a cost barrier to health care were not significantly associated with talking with a health care professional.

b Defined as limited in any way in any activities or use special equipment.

c Chronic diseases are arthritis, asthma, cardiovascular disease, cancer other than skin, chronic obstructive pulmonary disease, and diabetes.

d Determined from responses to 2 questions and included those who responded that, during the previous 12 months, they gave up household activities or chores they used to do because of confusion or memory loss that is happening more often or is getting worse always, usually, or sometimes (as opposed to rarely or never) or confusion or memory loss that is happening more often or is getting worse always, usually, or sometimes (as opposed to rarely or never) interfered with their ability to work, volunteer, or engage in social activities, or both.

The largest adjusted ORs for talking with a health care professional were for respondents who reported that SMCs always (vs never) caused them to give up household chores (OR, 3.02) or always (vs never) interfered with work (OR, 2.98) and for respondents who were college graduates (OR, 2.42) (Table 2). Compared with residents in California, residents in 7 states were more likely to talk to a health care professional. Others more likely to talk to a health care professional included respondents who had a recent check-up (compared with no recent check-up), had one or more personal doctors (compared with none), had a diagnosis of depression (compared with no such diagnosis), or had a disability (compared with no disability). The likelihood of talking with a health care professional decreased as age increased.

**Table 2:** Results of Logistic Regression Model^a^ for the Outcome Measure of Talking With a Health Care Professional About Memory Problems, Respondents Aged ≥45 Years Reporting Subjective Memory Complaints (N = 9,724^b^), BRFSS, 21 states, 2011

Group	Adjusted OR (95% CI)
**Age group, y**	
45–54	1.0 [Reference]
55–64	0.80 (0.60–1.05)
65–74	0.68 (0.49–0.93)
75–84	0.66 (0.46–0.95)
≥85	0.58 (0.36–0.95)
**Education**	
<High school	1.0 [Reference]
High school graduate	1.39 (0.98–1.99)
Some college	2.00 (1.39–2.87)
College graduate	2.42 (1.68–3.48)
**Had a routine check-up within the previous year**	
No	1.0 [Reference]
Yes	1.63 (1.20–2.21)
**Has ≥1 personal doctors**	
No	1.0 [Reference]
Yes	2.24 (1.41–3.57)
**Ever been diagnosed with depression**	
No	1.0 [Reference]
Yes	1.66 (1.30–2.13)
**Disability^c^**	
No	1.0 [Reference]
Yes	1.81 (1.38–2.37)
**Gave up household activities or chores**	
Never	1.0 [Reference]
Rarely	1.38 (0.98–1.93)
Sometimes	1.52 (1.13–2.06)
Usually	1.62 (0.93–2.82)
Always	3.02 (1.81–5.05)
**SMCs interfere with working, volunteering, or engaging in social activities**	
Never	1.0 [Reference]
Rarely	1.41 (1.02–1.95)
Sometimes	2.19 (1.60–3.01)
Usually	2.21 (1.38–3.53)
Always	2.98 (1.89–4.70)
**State of residence**	
California	1.0 [Reference]
Arkansas	1.29 (0.83–2.01)
Florida	1.43 (0.95–2.15)
Hawaii	1.83 (1.05–3.19)
Illinois	1.61 (1.00–2.58)
Iowa	1.49 (0.96–2.30)
Louisiana	1.85 (1.14–3.00)
Maryland	1.17 (0.65–2.10)
Michigan	1.33 (0.74–2.40)
Nebraska	1.03 (0.68–1.55)
New Hampshire	1.75 (1.07–2.85)
New York	1.51 (0.83–2.77)
North Carolina	1.35 (0.89–2.05)
Oklahoma	1.27 (0.81–1.98)
South Carolina	1.10 (0.69–1.73)
Tennessee	2.08 (1.21–3.59)
Texas	2.05 (1.34–3.12)
Utah	1.18 (0.67–2.07)
Washington	1.17 (0.80–1.71)
West Virginia	1.94 (1.22–3.08)
Wisconsin	1.22 (0.68–2.21)

Abbreviations: BRFSS, Behavior Risk Factor Surveillance System; CI, confidence interval; OR, odds ratio; SMC, subjective memory complaint.

^a ^The final logistic regression model excluded smoking, chronic diseases, body mass index, functional difficulties, and needing help due to SMCs because these variables were not found to be significant in the starting logistic model. The starting logistic regression model included all of the variables found in Table 1: age; education; disability; body mass index; chronic diseases; current smoking; check-up in previous year; one or more personal doctors; health status; ever diagnosed with depression; need help due to SMCs; gave up household chores or activities; SMCs interfere with work, volunteering, or engagement in social activities; functional difficulties; state of residence.

^b^ Sample size reflects the removal of 552 respondents for whom values for variables in the model were missing.

^c^ Defined as limited in any way in any activities or use special equipment.

Among respondents reporting SMCs who talked with a health care professional, 41.8% (95% CI, 37.2%–46.5%) received treatment, representing 9.5% of all adults aged 45 or older reporting SMCs. Treatment was more likely among respondents who had a diagnosis of dementia (74.3% [95% CI, 61.1%–84.1%]) than among those with SMCs who did not have a diagnosis of dementia (7.4% [95% CI, 6.2%–8.8%]). However, because so few reported a dementia diagnosis, among study respondents with SMCs who had been treated (n = 900), only 22.6% (95% CI, 17.2%–29.0%) reported a diagnosis of dementia.

Among respondents aged 65 or older reporting SMCs, 98.0% (95% CI, 97.1%–98.6%) had health insurance and 84.2% (95% CI, 82.0%–86.3%) reported a recent check-up. Among respondents aged 65 or older reporting SMCs, 17.9% (95% CI, 15.8%–20.3%) of those who had a recent check-up versus 11.9% (95% CI, 8.2%–17.0%) of those not reporting a recent check-up reported talking to a health care professional about their SMCs. Adults aged 65 or older reporting SMCs represent 23.3% (95% CI, 21.9%–24.7%) of respondents of all ages with SMCs.

## Discussion

These results agree with the results of previous studies (15–17) showing that few adults reporting memory problems are discussing them with a health care professional. The current results add to the body of information about such discussions in several ways. First, these results indicate that memory issues are often serious enough to affect daily activities such as household chores, work, or volunteering; however, even among those aged 45 or older most affected by SMCs, only about half reported such discussions. That the likelihood of such discussions decreased with increasing age indicates that even when adjusting for functional difficulties, older adults reporting SMCs are still less likely than adults aged 45 to 54 years to talk with a health care professional about them. This finding might have resulted from older adults feeling that their memory problems are a normal part of aging and nothing can be done about them, but the finding could have resulted from other causes. This finding also suggests that promoting assessments and discussions among adults younger than 65 might be beneficial. The increased likelihood of discussions among adults with disability and depression may indicate that adults reporting these conditions are more comfortable discussing issues with a health care professional or that these conditions create additional problems when combined with memory issues.

Having a recent check-up increased the likelihood of discussing memory problems with a health care professional, but only modestly (OR, 1.6). Even among those who had a recent check-up and reported that memory problems were affecting daily activities, only 37.4% had talked with a health care professional about their memory problems. Of course, any reported discussions could have taken place at some other time and with a different health care provider, but at least a routine check-up should have provided an opportunity for such a discussion. Although it is impossible to know what percentage of these adults reporting memory problems may progress to dementia or Alzheimer’s disease, a check-up could represent a missed opportunity for an assessment and discussion of available care, community services, and treatment options at their current stage of memory problems. Discussions could include a check for risk of falls, adherence with prescribed medications, review of exercise, smoking, dietary habits, level of social support, and consideration of future health care plans. Cognitive assessment tools have been developed (6) that could help in stimulating such discussions, and efforts are being made to raise awareness of the need for these activities (18). Because of the Affordable Care Act, those efforts currently focus on the Medicare population, estimated to include 93% of noninstitutionalized adults aged 65 or older (19). Based on these results for adults aged 65 or older with SMCs, there is room for improvement in discussing memory issues with a health care professional, although no information exists on adults who did not report SMCs. And, as mentioned above, results for adults younger than 65 suggest they might also benefit.

Also new in this study is that 41.8% of those who talked with a health care professional about their memory problems reported getting treatment. This percentage probably underestimates the percentage of those being treated because only those who said they had such discussions were asked that question, and some may be unaware that treatments they were receiving for other purposes also address memory problems. The data also provide no indication of when treatment occurred in relation to the discussion, what the treatment was, or if it was effective. However, the data suggest that at least in some cases the discussions might lead to treatment, especially because the treatments mentioned in the question were therapy or medications. The finding that those diagnosed with dementia were more likely than those not diagnosed to have been treated is not surprising given that the drugs currently available (20) treat Alzheimer’s and dementia rather than mild cognitive impairment. These drugs can temporarily help alleviate memory problems in some people who take them but cannot stop disease progression. New drugs in development may stop or slow the progression of the disease so that diagnosis at an earlier stage may be even more critical.

This study has several limitations. Results are subject to the usual limitations of telephone surveys, including possible bias due to nonresponse and errors caused by self-reporting and recall. Testing of other questions on the BRFSS suggests that the validity of measures in medical records (eg, a dementia diagnosis, recent check-up) is high (21), but the validity of the SMC measure is not known, nor has testing been done among respondents with cognitive impairment. People with dementia may be undiagnosed or unaware of their diagnosis, which might help explain respondents reporting treatment but not a diagnosis of dementia.

These results do not represent all noninstitutionalized adults because the BRFSS random selection process excludes a household if the selected respondent is physically or mentally unable to respond to the survey. A study using data from the cognitive impairment module in Wyoming (8) found that older nonrespondent adults in the household with SMCs for whom the respondent provided proxy responses were more likely to have functional difficulties (57% of proxied respondents vs 37% of respondents) and to have talked with a health care professional about their memory issues (60% of proxied respondents vs 23% of respondents) (8). Using cognitive impairment module data from 13 states (9), similar percentages were found for proxied responses only. Limiting studies to BRFSS respondents may be underestimating the extent of the impact of memory problems in the community. Another study limitation is the lack of information on respondents’ perceptions, beliefs, or family history of Alzheimer’s or dementia, factors that may affect discussions with health care providers (15,16). The generalizability of the results is unknown because of the state-by-state variation in these results and those reported elsewhere (7).

This study has several strengths. The BRFSS provides a large and representative sample; this study included more than 10,000 adults in 21 states reporting memory problems, and more than 2,200 reported talking with a health care professional about these problems. The study included a large number of variables, including information on demographics, self-reported behaviors, chronic conditions, access to health care, and health status.

These findings suggest that routine check-ups are a missed opportunity for assessing and discussing memory problems for the majority of adults aged 45 or older with SMCs. These results, based only on adults reporting SMCs, highlight the need for the cognitive assessment required in the Affordable Care Act for Medicare recipients and also suggest the need and potential benefits of cognitive assessment among adults younger than 65. Although memory problems could be discussed during any routine check-up, these results show that few adults reporting SMCs, even those with memory problems causing functional difficulties, are availing themselves of that opportunity. Public health officials and organizations can become involved by raising general awareness of the advantages of early detection of cognitive impairment and dementia and discussions with a health care professional. An example of such a message to health care providers is a letter from the New York State Department of Health that includes information from the Alzheimer’s Association on conducting assessments during the Medicare annual wellness visit (22). These actions could potentially result in greater numbers of older adults being diagnosed and treated for memory problems and made aware of available services.

## Figures and Tables

**Table:** 

**1. The activity supported the learning objectives.**				
**Strongly Disagree**				**Strongly Agree**
1	2	3	4	5
**2. The material was organized clearly for learning to occur.**				
**Strongly Disagree**				**Strongly Agree**
1	2	3	4	5
**3. The content learned from this activity will impact my practice.**				
**Strongly Disagree**				**Strongly Agree**
1	2	3	4	5
**4. The activity was presented objectively and free of commercial bias.**				
**Strongly Disagree**				**Strongly Agree**
1	2	3	4	5

## Post-Test Information

To obtain credit, you should first read the journal article. After reading the article, you should be able to answer the following, related, multiple-choice questions. To complete the questions (with a minimum 75% passing score) and earn continuing medical education (CME) credit, please go to http://www.medscape.org/journal/pcd. Credit cannot be obtained for tests completed on paper, although you may use the worksheet below to keep a record of your answers. You must be a registered user on Medscape.org. If you are not registered on Medscape.org, please click on the "Register" link on the right hand side of the website to register. Only one answer is correct for each question. Once you successfully answer all post-test questions you will be able to view and/or print your certificate. For questions regarding the content of this activity, contact the accredited provider, CME@medscape.net. For technical assistance, contact CME@webmd.net. American Medical Association's Physician's Recognition Award (AMA PRA) credits are accepted in the US as evidence of participation in CME activities. For further information on this award, please refer to http://www.ama-assn.org/ama/pub/about-ama/awards/ama-physicians-recognition-award.page. The AMA has determined that physicians not licensed in the US who participate in this CME activity are eligible for ***AMA PRA Category 1 Credits*** ™. Through agreements that the AMA has made with agencies in some countries, AMA PRA credit may be acceptable as evidence of participation in CME activities. If you are not licensed in the US, please complete the questions online, print the AMA PRA CME credit certificate and present it to your national medical association for review.

## Post-Test Questions

### Study Title: Routine Check-Ups and Other Factors Affecting Discussions With a Health Care Provider About Subjective Memory Complaints, Behavioral Risk Factor Surveillance System, 21 States, 2011

**CME Questions**

1. Your patient is a 62-year-old man with subjective memory complaints (SMCs). According to the surveillance study by Adams, which of the following statements about the percentage of older adults with SMCs who discussed memory problems with their clinician is *correct*?
  - Among all respondents at least 45 years old who reported SMCs, more than half reported discussing them with a health care professional
  - Among all respondents at least 45 years old who reported SMCs and had a recent routine check-up, more than two-thirds reported discussing them with a health care professional
  - Among respondents 65 years or older reporting SMCs, 17.9% of those who had a recent check-up, vs 11.9% of those who did not, reported talking to a health care professional about their SMCs
  - The results differed considerably from those of previous studies
2. According to the surveillance study by Adams regarding older adults with SMCs, which of the following variables is *most* likely associated with these patients discussing their memory problems with their clinician?
  - Reporting that SMCs never caused them to give up household chores
  - Reporting that SMCs never interfered with work
  - Increasing age
  - Being a college graduate
3. According to the surveillance study by Adams, which of the following statements about the clinical implications regarding older adults with SMCs discussing their memory problems with their clinician is *correct*?
  - Among respondents who discussed SMCs, one-quarter received treatment
  - Routine check-ups may be a missed opportunity for discussions of SMCs that might lead to diagnosis or treatment
  - The Affordable Care Act requires a cognitive assessment for Medicare recipients every 5 years
  - Among study respondents with SMCs who received treatment, three-quarters reported a diagnosis of dementia

**Evaluation**
